# Genome-Wide Identification and Evolution of Receptor-Like Kinases (RLKs) and Receptor like Proteins (RLPs) in *Brassica juncea*

**DOI:** 10.3390/biology10010017

**Published:** 2020-12-30

**Authors:** Hua Yang, Philipp E. Bayer, Soodeh Tirnaz, David Edwards, Jacqueline Batley

**Affiliations:** 1 School of Biological Sciences, University of Western Australia, Crawley, WA 6009, Australia; hua.yang1@uq.net.au (H.Y.); Philipp.bayer@uwa.edu.au (P.E.B.); soodeh.tirnaz@research.uwa.edu.au (S.T.); Dave.edwards@uwa.edu.au (D.E.); 2 School of Agriculture and Food Sciences, University of Queensland, St Lucia, QLD 4067, Australia

**Keywords:** receptor-like kinases (RLK), receptor-like proteins (RLP), disease resistance, Indian mustard, *Brassica juncea*, resistance genes

## Abstract

**Simple Summary:**

Plants have evolved defence mechanisms to protect themselves against microbial pathogens. The identification of genes underlying quantitative trait loci is extremely challenging in complex polyploid genomes. In this research, we identify and characterise two types of resistance genes; RLKs (receptor like kinases) and RLPs (receptor like proteins) in Indian mustard (*Brassica juncea*), one of the major crops in India and an important member of the Brassicaceae family, which can be linked to QTL for disease resistance. The outcome provides a valuable resource for facilitating the identification of functional resistance genes which can be employed by breeders toward the production of resistant cultivars.

**Abstract:**

*Brassica juncea*, an allotetraploid species, is an important germplasm resource for canola improvement, due to its many beneficial agronomic traits, such as heat and drought tolerance and blackleg resistance. Receptor-like kinase (RLK) and receptor-like protein (RLP) genes are two types of resistance gene analogues (RGA) that play important roles in plant innate immunity, stress response and various development processes. In this study, genome wide analysis of RLKs and RLPs is performed in *B. juncea*. In total, 493 RLKs (LysM-RLKs and LRR-RLKs) and 228 RLPs (LysM-RLPs and LRR-RLPs) are identified in the genome of *B. juncea*, using RGAugury. Only 13.54% RLKs and 11.79% RLPs are observed to be grouped within gene clusters. The majority of RLKs (90.17%) and RLPs (52.83%) are identified as duplicates, indicating that gene duplications significantly contribute to the expansion of RLK and RLP families. Comparative analysis between *B. juncea* and its progenitor species, *B. rapa* and *B. nigra*, indicate that 83.62% RLKs and 41.98% RLPs are conserved in *B. juncea*, and RLPs are likely to have a faster evolution than RLKs. This study provides a valuable resource for the identification and characterisation of candidate RLK and RLP genes.

## 1. Introduction

In plants, cell surface receptors play an important role in perceiving self-derived or non-self-derived extracellular signals, where the communication between the extracellular matrix and the cell interior happens [1,2]. Plant receptor-like kinases (RLKs) and receptor-like proteins (RLPs) constitute two major classes of cell-surface receptors. Generally, the plant cell surface receptors participate in innate immunity, stress responses and a wide variety of developmental processes [3,4,5,6,7,8,9]. A typical structure of RLKs is composed of a single-pass transmembrane domain and a cytoplasmic kinase domain [10,11]. RLPs are structurally similar to RLKs. The only difference is that the RLP carries a short cytoplasmic tail instead of the intracellular kinase domain present in RLKs [9,12]. Due to the kinase domain, the RLKs can trigger signalling on their own, whereas RLPs require the formation of a functional complex with an RLK to activate downstream signalling [13,14,15]. The first RLP was identified in *Solanum lycopersicum*; defined as *Cf-9*, a leucine-rich repeat RLP (LRR-RLP), which confers resistance to the effector *Avr9* of the fungus *Cladosporium fulvum* [16]. Whereas, the first report of a RLK, *ZmPK1*, was isolated from *Zea mays*, and belongs to the S-domain class, functioning in specific self-pollen recognition [17].

RLKs and RLPs can be classified into sub-families on the basis of the type of extracellular domain they carry. The largest subfamily of RLK and RLP families carry a leucine-rich repeat (LRR) domain and are defined as LRR-RLK and LRR-RLP [3,4]. These two sub-families, LRR-RLKs and LRR-RLPs, are known to participate widely in plant defence and development. In plants, both LRR-RLKs and LRR-RLPs have been observed to contain significant rates of duplication, loss or retention [10,12,18,19,20,21,22].

Further sub-families of RLKs and RLPs carry the Lysin motif (LysnM) and are known as LysM-RLK and LysM-RLP. These sub-families of proteins play an important role in innate immunity and/or symbiosis. In *Arabidopsis thaliana*, the LysM-containing chitin elicitor RLK1 (CERK1) has been found to be essential for chitin perception and also functions in defence response against fungal pathogens [23,24,25,26]. In contrast, LysM-RLKs in legumes coordinate with rhizobial bacteria to control symbiotic processes, through perceiving secreted bacterial lipochitooligosaccharides (Nod factor, NF) signals [27,28,29,30,31].

*Brassica juncea*, commonly known as Indian mustard, is an allotetraploid plant species with AABB genome produced by natural interspecific hybridisation between the diploid species *B. nigra* (BB) and *B. rapa* (AA) [32,33]. It is widely planted, as it is known to contain many beneficial agronomic traits compared to *B. napus*, such as increased heat and drought tolerance, high blackleg and shatter resistance and early vigour [34,35,36,37,38,39,40]. The *B. juncea* reference genome, 922 Mb in length, is currently available [33]. This provides an opportunity to characterise the LRR-RLK and LRR-RLP genes in *B. juncea* and understand their genomic distribution.

Whilst a genome-wide study of the evolution and distribution of nucleotide binding site-leucine-rich repeat (NLR) resistance genes have been conducted in *B. juncea* [41], the majority of RLK (LysM-RLK and LRR-RLK) and RLP (LysM-RLP and LRR-RLP) genes remain uncharacterised in *B. juncea*. Here, we present the identification, characterisation and distribution of the RLKs and RLPs subfamilies in the *B. juncea* genome. Comparative analyses are also performed to identify the orthologous genes and define collinear relationships between *B. juncea* and its related diploid progenitors, *B. rapa* and *B. nigra*. The outcomes provide information that facilitate the further investigation of resistance gene evolution and distribution in *B. juncea*, which ultimately can assist breeders for resistance improvement.

## 2. Materials and Methods

### 2.1. Brassica Reference Genomes

The Brassica genomes used in this study, include *B. juncea* T84-66 v1.5 (genome: AABB, 779.5 Mbp) [33], *B. nigra* DH YZ12151 scaffold version (genome: BB) [33] and *B. rapa* Chiifu-401-42 v2.5 (genome: AA, 370.2 Mbp) [42]. Their genome sequence, annotation and protein sequences were obtained from the website http://brassicadb.org/brad/.

### 2.2. Genome Wide Identification of RLK and RLP Genes

Candidate proteins of RLK and RLP families were retrieved using the RGAugury pipeline [43]. Firstly, BLASTp analysis was performed to screen potential RGA candidates in *Brassica* species against the database RGAdb with an E-value cut-off of E^−5^. Secondly, specific domains in RLKs (LRR, LysM, TM and STTK domains) and RLPs (LRR, LysM and TM domains) were detected through InterProScan and Phobius. Finally, the genes were classified into RLKs and RLPs on the basis of their unique domain structures. *Brassica* RLKs were grouped into three-types: LRR-RLK, LysM-RLK and RLK-other-receptor; while RLPs contained two types, namely LRR-RLP and LysM-RLP. In this study, we focused on LysM and LRR types of RLKs and RLPs.

### 2.3. Genomic Distribution of RLK and RLP Genes

RLKs and RLPs were mapped to their corresponding chromosomes on the basis of their physical positions in the *Brassica* genomes. Gene clusters were determined using the physical positions of the genes which were situated on the same chromosome. In this study, a gene cluster was defined as a chromosome region harbouring three or more genes within 200 kb, according to previous literature [44]. Clusters formed by tandem duplications were defined as a tandem cluster. RLK and RLP clusters were detected, according to the kinase domain.

### 2.4. Gene Duplication Analysis of RLK and RLP Genes

Gene duplication analysis in this study was performed on the basis of previously reported criteria, with over 70% in both the coverage and identity of alignment during BLASTp comparison [45,46,47]. Gene duplicates located within a 5 Mb region on the same chromosome were considered as tandemly duplicated, while those situated beyond 5 Mb were defined as segmentally duplicated [48,49,50]. The duplication events observed within the *B. juncea* A or B sub-genomes were defined as intra-genomic duplications. In contrast, inter-genomic duplications referred to the duplications between the A and B sub-genomes.

### 2.5. Ortholog and Paralog Analysis

Paralogous copies were defined as the duplicated genes which occupied two different positions in the same genome [51,52]. Paralogous genes were confirmed by the BLASTp comparison of all the predicted RLKs and RLPs proteins against each other with E-value of E-20 [45]. However, orthologous genes, which were observed in two different species originating from a common ancestor, were confirmed when the E-value was smaller than E-45 during BLASTp comparison of all the predicted RLK or RLP proteins against each other, with over 70% of similarity and coverage [51,52].

### 2.6. Multiple Alignment and Phylogenetic Analysis

Multiple sequence alignment of RLK and RLP proteins were performed separately in Geneious v7.1.9 with the CLUSTAL W method (Biomatters, Auckland, New Zealand) [53]. The neighbour-joining method with 1000 bootstrap iterations was used to construct a phylogenetic tree with aligned proteins in Geneious v7.1.9 (Biomatters, Auckland, New Zealand) [53].

## 3. Results

### 3.1. Genome-Wide Identification of RLK and RLP Genes in B. juncea

A total of 493 candidate RLK genes, accounting for 0.613% of the predicted coding genes, were identified in the *B. juncea* genome, which is over twice as many as the RLP family of 228 genes (0.283%) (Table 1). Only nine LysM-RLKs and two LysM-RLPs were observed in *B. juncea*, suggesting that the LysM subfamily was much smaller than the LRR subfamily, which contained 484 LRR-RLKs (98.17% of RLKs) and 226 LRR-RLPs (99.12% of RLPs), respectively (Table 1).

### 3.2. Genomic Distribution of RLK and RLP Genes in B. juncea

**Figure 1:**
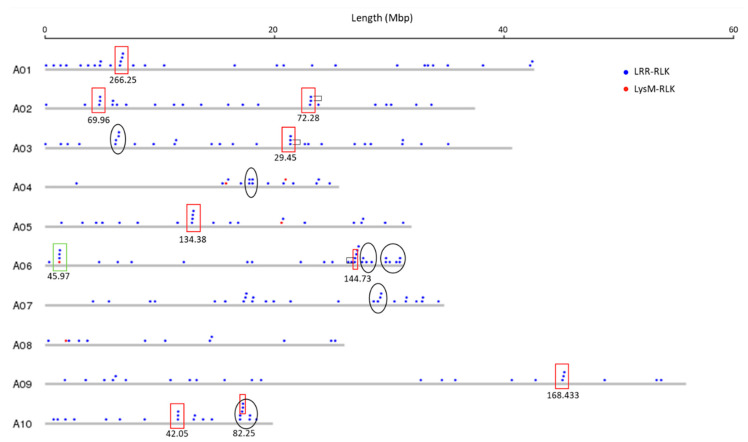
Physical location of receptor-like kinase (RLK) genes (LysM-RLK and LRR-RLK) on the chromosomes of the A genome of *B. juncea*. Solid circles above the chromosome (grey bars) represent RLK genes: LysM-RLK (red) and LRR-RLK (blue). Rectangles represent gene clusters: homogeneous cluster (red) and heterogeneous cluster (green). Numbers below the rectangles indicate the size of gene cluster in kb. Unfilled black ellipses indicate regions rich in RLK genes. Black brackets indicate tandemly-duplicated genes within a gene cluster. Chromosome lengths are displayed in Mbp (megabase pairs) on the scale at top.

**Figure 2:**
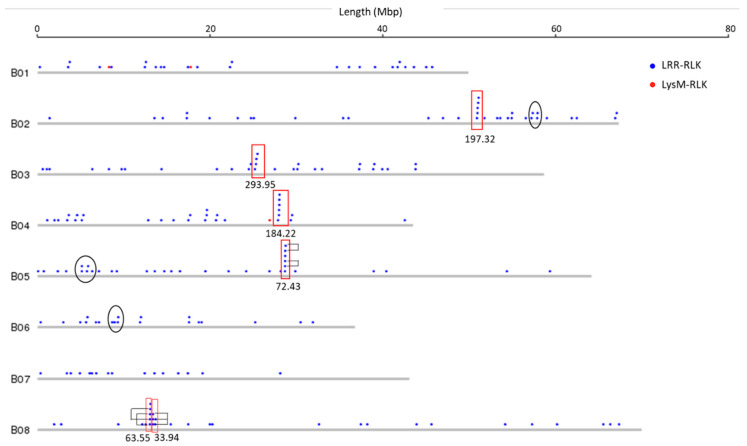
Physical location of RLK genes (LysM-RLK and LRR-RLK) on the chromosomes of the B genome of *B. juncea*. Solid circles above the chromosome (grey bars) represent RLK genes: LysM-RLK (red) and LRR-RLK (blue). Red rectangles represent gene clusters. Numbers below the rectangles indicate the size of gene cluster in kb. Unfilled black ellipses indicate regions rich in RLK genes. Black brackets indicate tandemly-duplicated genes within a gene cluster. Chromosome lengths are displayed in Mbp (megabase pairs) on the scale at top.

**Figure 3:**
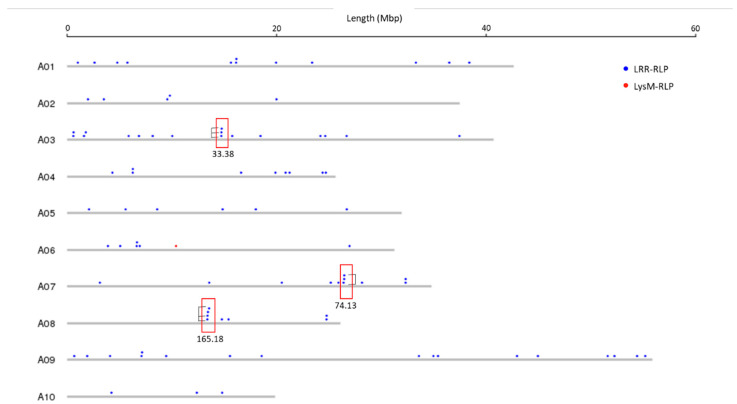
Physical location of receptor-like protein (RLP) genes (LysM-RLP and LRR-RLP) on the chromosomes of the A genome of *B. juncea*. Solid circles above the chromosome (grey bars) represent RLP genes: LysM-RLP (red) and LRR-RLP (blue). Red rectangles represent the gene cluster. Numbers below the rectangles indicate the size of gene cluster in kb. Black brackets indicate tandemly-duplicated genes within a gene cluster. Chromosome lengths are displayed in Mbp on the scale at top.

**Figure 4:**
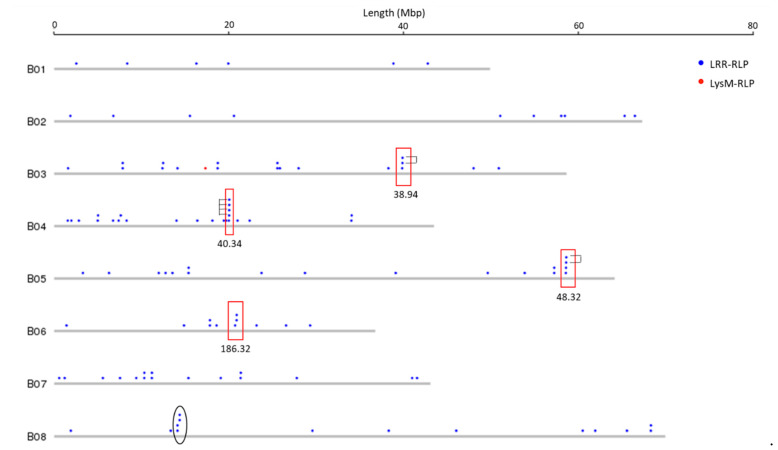
Physical location of RLP genes (LysM-RLP and LRR-RLP) on the chromosomes of the B genome of *B. juncea*. Solid circles above the chromosome (grey bars) represent RLP genes: LysM-RLP (red) and LRR-RLP (blue). Red rectangles represent the gene cluster. Numbers below the rectangles indicate the size of gene cluster in kb. Unfilled black ellipses indicate regions rich in RLP genes. Black brackets indicate tandemly-duplicated genes within a gene cluster. Chromosome lengths are displayed in Mbp (megabase pairs) on the scale at top. In *B. juncea*, the mapped RLKs for each chromosome ranged from 2.62% (12 genes on chromosome A08) to 7.86% (36 genes on B02) (Table S1). However, 10.85% (23 genes) of the assigned RLPs were found to be located on chromosome B04, followed by 19 genes (8.96%) on B03, and the least number were observed on A10 with three genes (1.42%) (Table S2). In addition, the gene number of both RLKs and RLPs was found to be higher on four chromosomes (B03 to B05, and B08), compared with the other chromosomes (Tables S1 and S2).

Physical mapping was performed to detect the distribution of candidate RLK and RLP genes across the *B. juncea* genome (Figure 1, Figure 2, Figure 3 and Figure 4; Tables S1 and S2). In total, 92.90% of RLKs (458 genes) and 92.98% of RLPs (212 genes) were physically mapped, according to their corresponding positions in the genome. The remaining genes (35 RLKs and 16 RLPs) could not be assigned to any chromosome in *B. juncea*.

In *B. juncea*, the assigned RLKs were distributed almost evenly between the A and B genomes, with 236 (51.53%, five LysM-RLKs and 231 LRR-RLKs) and 222 (48.47%, three LysM-RLKs and 219 LRR-RLKs) (Table S1). The number of RLP genes (117) in the B genome was slightly higher than in the A genome, which contained 95 genes (Table S2). All of the assigned candidate genes were found to be distributed across all the 18 chromosomes in *B. juncea* (Figure 1, Figure 2, Figure 3 and Figure 4). LRR-RLKs and LRR-RLPs could be observed on each chromosome, while LysM-RLKs were only present on some chromosomes (A04–A06, A08, B01 and B04). However, only one LysM-RLP was situated on chromosome A06 and B03 (Figure 1, Figure 2, Figure 3 and Figure 4; Table S2).

### 3.3. RLK and RLP Gene Clustering in B. juncea

In this study, a gene cluster was defined as a chromosomal region harbouring three or more genes within 200 kb. Gene cluster analysis was performed for RLKs and RLPs. Accordingly, 13.54% (62 genes) of the assigned RLKs were located in gene clusters, while only 25 RLPs (11.79%) were observed in RLP clusters (Tables S1 and S2). Most of the assigned RLKs and RLPs represented a single-gene locus in *B. juncea*. In addition, un-clustered RLKs and RLPs were located across the genome of *B. juncea* (Figure 1, Figure 2, Figure 3 and Figure 4). Some of the un-clustered RLKs and RLPs were closely situated in the chromosomes, forming regions rich in RLKs and RLPs (Figure 1, Figure 2, Figure 3 and Figure 4).

In total, 16 RLK clusters were observed in the *B. juncea* genome, where 10 of them resided in the A genome, more than in the B genome with six gene clusters (Figure 1 and Figure 2; Table S1). The RLK clusters were almost evenly distributed in the chromosomes of *B. juncea*, except for on six chromosomes (A04, A07, A08, B01, B06 and B07) (Table S1). However, there were only seven RLP gene clusters, which were evenly distributed in seven chromosomes of the *B. juncea* genome, with three in the A genome, less than in the B genome which had four clusters (Figure 3 and Figure 4; Table S2). All of the RLP clusters were homogeneous, since LysM-RLP genes were not involved in generating gene clusters (Figure 3 and Figure 4; Table S2). By contrast, one LysM-RLK gene, located on chromosome A06, participated in the formation of RLK clusters, resulting in the presence of a heterogeneous cluster (Figure 1 and Figure 2; Table S1).

Most of the large RLK clusters were situated in the B genome chromosomes of *B. juncea*, where the largest clusters containing six RLKs were found on chromosomes B04 and B05, followed by clusters of five genes on B02 and B08 (Figure 2; Table S1). Additionally, the average gene number of a RLK cluster in the A genome was three genes, smaller than in the B genome with five genes (Table S1). Contrastingly, the highest gene number in a RLP cluster, having five genes, was observed on chromosome B04 (Figure 3 and Figure 4; Table S2). Most of the RLP clusters, which were located on chromosomes A03, A07, B03 and B06, contained three genes (Figure 3 and Figure 4; Table S2).

The size of a cluster was determined by the sequence length between the two RLKs or RLPs situated at both ends of the gene cluster. The size of RLK clusters, with an average of 118.82 kb, ranged from 29.45 kb on A03 to 293.95 kb on chromosome B03 (Figure 1 and Figure 2). Similarly, RLP cluster size also ranged from 33.38 kb to 186.32 kbp on chromosomes A03 and B06, respectively, compared with the average size of 83.80 kb (Figure 3 and Figure 4). The size of RLK and RLP clusters was not related to the gene number in the cluster (Figure 1, Figure 2, Figure 3 and Figure 4).

### 3.4. Analysis of Duplications and Paralogues of RLK and RLP Genes in B. juncea

**Figure 5:**
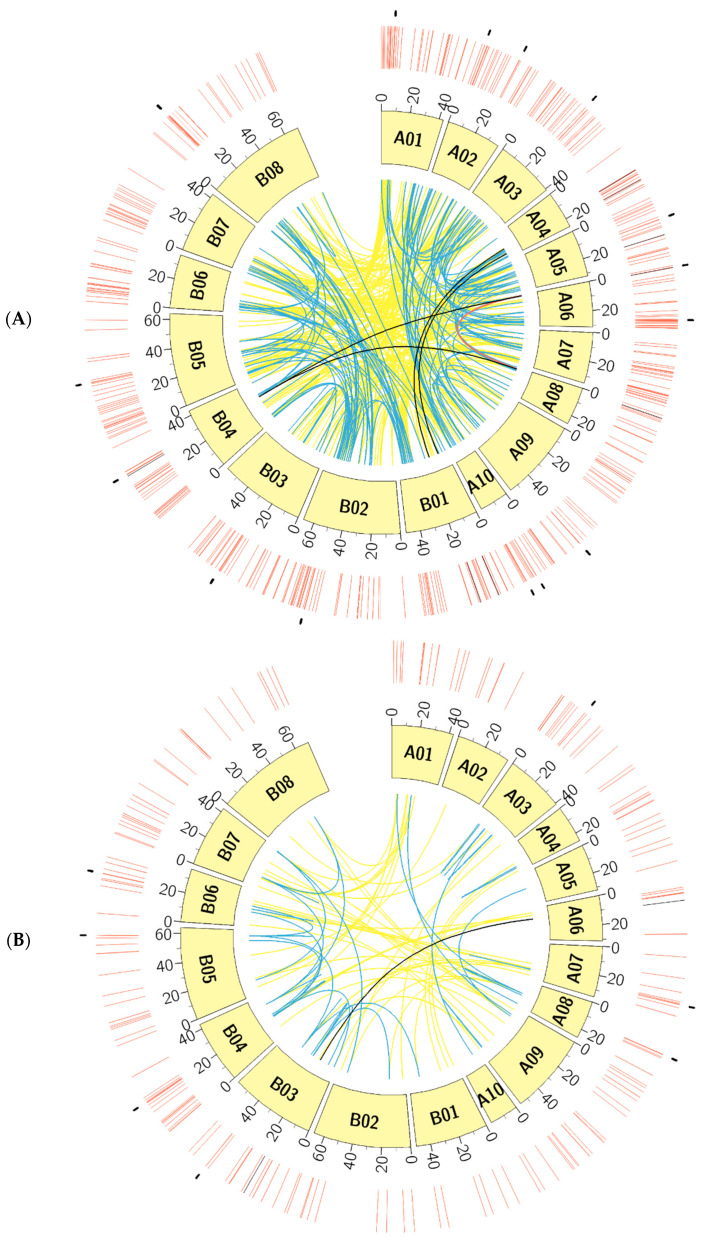
Chromosomal distribution, organisation and duplication of 458 candidate RLK genes (**A**) and 212 candidate RLP *genes* (**B**) in the *B. juncea* genome.

In this study, duplicated genes were detected using BLASTp by comparing all predicted proteins against each other. The RLK and RLP gene families were analysed separately. Duplicated genes, which occupy two different positions in the same genome, were defined as paralogous copies [51,52]. As *B. juncea* is an allotetraploid species, [32,33], two types of genomic duplications were observed; intra- and inter-genomic duplications (Figure 5; Table 2, Tables S1 and S2). The former is where duplication occurs within the A or B sub-genome of *B. juncea*. By contrast, the duplication events that occur between the A and B sub-genomes are considered as inter-genomic duplications (Figure 5).

A total of 413 RLKs (90.17% of the mapped RLKs), consisting of seven LysM-RLKs and 406 LRR-RLKs, were found to be involved in duplication events. Accordingly, 413 paralogous copies were formed in the *B. juncea* genome (Table S1). A total of 52.83% of the assigned RLPs were defined as duplications, with 112 genes including two (1.79%) LysM-RLPs and 110 (98.21%) LRR-RLPs. There were 112 RLP paralogous copies in the *B. juncea* genome (Table S2). In *B. juncea*, most of the assigned RLK genes (90.17%) had more than one copy, while approximately half of the mapped RLPs (47.17%) were a single copy.

The outer circle illustrates the position of RLK/RLP clusters. The LysM-RLK/RLP (black) and LRR-RLK/RLP (red) type genes were mapped on 18 chromosomes (A01–A10, B01–B08) in the inner circle. The coloured lines in the centre represent the duplication events between chromosomes: inter-genomic duplications of LRR-RLKs/RLPs (yellow) and LysM-RLKs/RLPs (black); intra-genomic duplication events of LRR-RLKs/RLPs (blue) and LysM-RLKs/RLPs (red).

Segmental duplications made minor contributions to RLK and RLP duplications. However, tandem duplications play a major role in RLP duplications compared to RLK duplications. In the duplicated RLK genes, 14.04% (58 genes) were derived from tandem duplications, while only 3.87% (16 genes) were from segmental duplications (Table S1). For the RLPs, 46.43% (52 genes) of the duplicated genes resulted from tandem duplication, while only 3.57% (4 genes) were from segmental duplications (Table S2).

The duplicated RLKs were mostly evenly distributed in the A and B sub-genomes in *B. juncea*, with 209 and 204 genes, respectively, consistent with the genomic distribution of duplicated RLPs, with 52 and 60 genes, respectively (Tables S1 and S2). However, these duplicated RLKs and RLPs were unevenly distributed over the 18 chromosomes of *B. juncea* (Figure 5; Tables S1 and S2). Additionally, the highest number of duplicated RLKs was found on chromosome B02, containing 34 LRR-RLKs, followed by B05 with 32 LRR-RLKs (Table S1). All of the LRR-RLKs residing on chromosomes A05 (21) and B07 (16) were involved in duplication events (Table S1). Chromosomes B03 and B04 had the highest number of duplicated RLPs, each containing 11 genes, while the least number (one) was found on chromosomes A02, A05 and A10 (Table S2).

In total, 82% (51 genes) of the clustered RLKs were found to be duplicated, as well as 88% (22 out of 25 genes) of clustered RLPs (Tables S1 and S2). Six out of 16 RLK clusters were formed by tandem duplication, while almost all of the RLP clusters (six out of seven) were defined as tandem clusters (Figure 1, Figure 2 and Figure 3; Tables S1 and S2). For instance, the gene cluster on chromosome A02 contained three LRR-RLK genes, of which two were duplicates. Another example was the cluster on chromosome B08, constructed with three tandem duplicates.

A total of 471 and 110 duplication events were detected in the RLK and RLP families, respectively (Table 2). For both the RLK and RLP families, almost all of the duplication events occurred in the LRR sub-family, with 466 in LRR-RLKs and 109 in LRR-RLPs, in sharp contrast with five in LysM-RLKs and one in LysM-RLPs (Table 2).

Two types of duplication events, inter and intra duplications, were observed in both *B. juncea* RLK and RLP families (Table 2). For *B. juncea* RLKs, the number of inter-genomic duplications was much higher than intra-genomic, with 308 and 163, respectively. For the RLP family, the number of these two types of duplications was smaller, with 52 intra-genomic duplications and 58 inter-genomic duplications (Table 2). Furthermore, the intra-genomic duplications of RLKs were evenly distributed in *B. juncea* A and B genomes, with 83 and 80, respectively, while the RLP duplication events in the A genome (20) were less than in the B genome of 32 (Table 2). Almost all of the LysM-RLK duplications (four out of five) were defined as inter-genomic duplications, and in LysM-RLP, there was only one duplication event found between the A and B sub-genomes (Table 2). These two kinds of duplications for RLKs could be observed in all 18 chromosomes of *B. juncea* (Figure 5; Table S1). For RLPs, the B sub-genome contained both inter- and intra- genomic duplication events, while in the A sub-genome, the chromosomes A02 and A06 did not have intra-genomic duplication and A05 and A10 did not have the inter-genomic type (Table S2). The duplicated events could be observed across the *B. juncea* genome (Figure 5).

### 3.5. Phylogenetic Analysis of RLK and RLP Genes in B. juncea

The evolutionary relationship between predicted proteins was studied by the construction of phylogenetic trees, using the neighbour-joining (NJ) method with 1000 bootstrap replicates (Figures S1 and S2). Here, the RLKs and RLPs were analysed separately.

A total of 493 RLKs were divided into seven major groups, which were further classified into subgroups and clades (Figure S1). However, the size of the groups significantly varied, ranging from four in Group VI to 145 in Group I, with an average of 70 genes in each group. Additionally, all of the LysM-RLKs displayed a close relationship with some LRR-RLKs in Group I. In the phylogenetic tree, the predicted paralogous RLKs were grouped together with high bootstrap value. The majority of subgroups or clades consisted of paralogous genes located on the same chromosome or homeologous chromosomes, which were derived from duplications events. For example, LysM-RLK genes (BjuA002911 on chromosome A08, BjuA021332 on A06, and BjuB004835 on B04), defined as paralogous genes, were grouped together in Group I.

In total, 228 RLPs were classified into three primary groups through phylogenetic analysis (Figure S2). Similar to LysM-RLKs, LysM-RLPs were also closely related to some LRR-RLPs in Group III (Figure S2). Differently from RLKs, many branches were formed with a single RLP gene, suggesting the distant evolutionary relationship and the functional diversity among RLP genes (Figure S2).

### 3.6. Comparison and Conservation Analysis of RLK and RLP Genes between B. juncea and Its Diploid Progenitor Species B. rapa and B. nigra

*B. juncea*, an allotetraploid species (AABB), was generated through interspecific hybridisation between two ancestral diploid genomes of *B. rapa* (AA) and *B. nigra* (BB) [32,33]. Genomic comparison of RLKs and RLPs is important to understand the genetic diversity and evolution between these *Brassica* species.

In total, 493 RLKs and 228 RLPs were identified in *B. juncea*, of which 236 RLKs and 95 RLPs were located in the A genome, and the remaining genes resided in the B genome (Tables S1 and S2). In *B. rapa*, genome-wide identification indicated that 0.651% (300 genes) of the total predicted genes were detected as RLK genes, consisting of three LysM-RLKs and 297 LRR-RLKs, which is higher than in the *B. juncea* A genome (Table 1 and Table 3). Conversely, 65 RLPs (0.141%) in *B. rapa*, which included two LysM-RLPs and 63 LRR-RLPs, were less than those found in the *B. juncea* A genome with 95 genes (Table 1 and Table 3). A total of 317 RLK genes (0.636% of the total predicted genes) were identified from the *B. nigra* scaffold sequence, with five LysM-RLKs and 312 LRR-RLKs more than in the B genome of *B. juncea*. Similarly, the number of RLPs (0.353%) in *B. nigra* was also higher than in *B. juncea*, with 176 and 117 genes, respectively (Table 1, Table 4 and Table 5).

In these three *Brassica* species, the proportions of RLK genes were similar (around 0.613% to 0.651%), while those of RLPs varied from 0.141% to 0.353% (Table 2). Additionally, LysM and LRR subgroups could be observed in each RLK and RLP family, while the percentage of the LysM subfamily was found to be extremely low, below 4%, in both RLK and RLP families (Table 3, Table 4 and Table 5).

**Figure 6:**
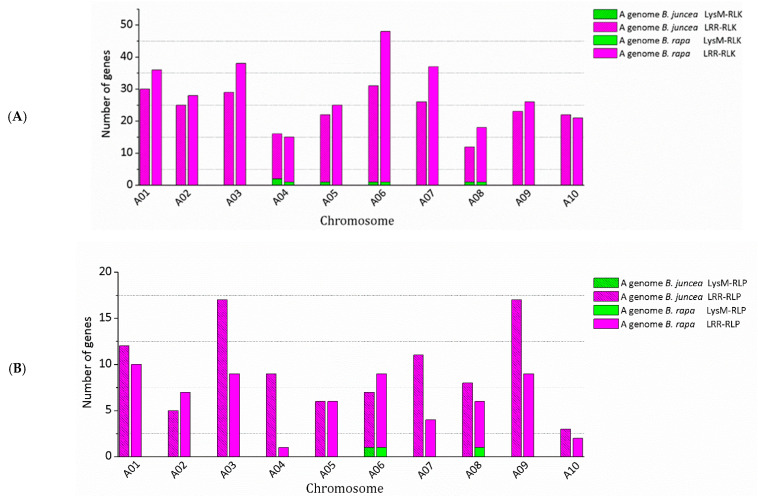
Distribution of candidate RLK (**A**) and RLP (**B**) genes in the A genome of *B. juncea* and *B. rapa*.

The chromosomal distribution and clustering of RLK and RLP genes were not analysed in B. nigra, as a good quality assembled genome was not available. Genes of the RLK family in the A genome of B. juncea shared a similar distribution pattern with *B. rapa* in gene number and distribution (Figure 6; Table 3 and Table 4). For instance, chromosome A06 in *B. juncea* and *B. rapa* contained the highest number of RLKs, and the least number was observed in A04 and A08. Additionally, the chromosomal distribution pattern of the LRR-RLK subfamily was consistent with the RLK family in the A genomes of *B. juncea* and *B. rapa* (Figure 6; Table 3 and Table 4). However, the distribution of LysM-RLK genes was slightly different between these two *Brassica* species, as the presence of LysM-RLK on chromosome A05 of *B. juncea* was not observed in *B. rapa* (Figure 6; Table 3 and Table 4).

The chromosomal distribution of RLPs differed between the A genome of *B. juncea* and *B. rapa* (Figure 6; Table 3 and Table 4). For instance, the highest number (17 genes) of RLPs were found on chromosomes A03 and A09 in the A genome of *B. juncea*, while it was observed on A01 and A06 of *B. rapa*, with 10 genes. Furthermore, a different distribution pattern could be observed in both the LRR-RLP or LysM-RLP subfamilies between the A genome of these two *Brassica* species (Figure 6; Table 3 and Table 4). For example, the LysM-RLP gene, lost in *B. juncea* A08 was present in *B. rapa*.

**Figure 7:**
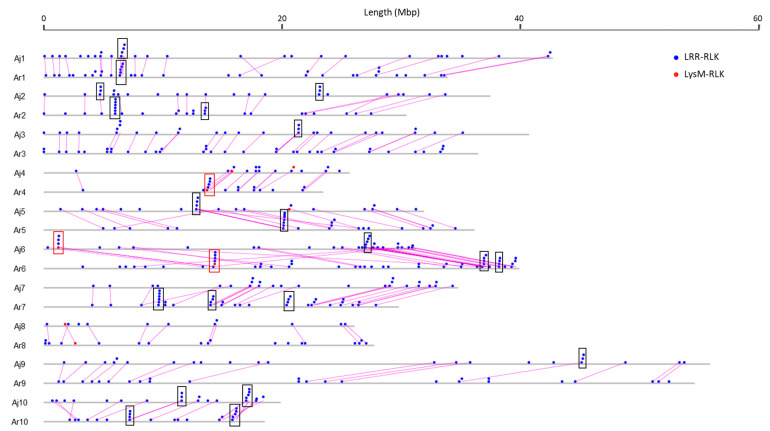
Comparative organisation of RLK genes between Aj genome of *B. juncea* and Ar genome of *B. rapa* (chromosomes A01 to A10). Solid circles above the chromosome (grey bars) represented RLK genes: LysM-RLK (red) and LRR-RLK (blue). Rectangles represented gene clusters: homogeneous cluster (black) and heterogeneous cluster (red). Pink straight lines between genes displayed the genes with syntenic relationship between *B. juncea* and *B. rapa*. Chromosome lengths were displayed in Mbp (megabase pairs) on the scale at top.

**Figure 8:**
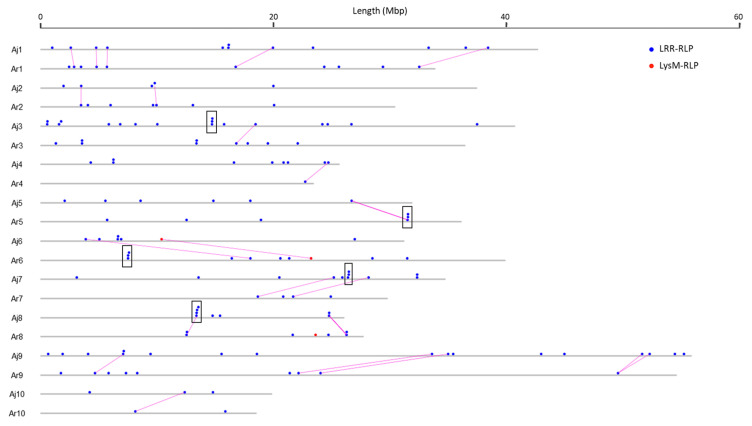
Comparative organisation of RLP genes between Aj genome of *B. juncea* and Ar genome of *B. rapa* (chromosomes A01 to A10). Solid circles above the chromosome (grey bars) represented *RLK* genes: LysM-RLP (red) and LRR-RLP (blue). Black rectangles represented homogeneous cluster. Pink straight lines between genes displayed the genes with syntenic relationship between *B. juncea* and *B. rapa*. Chromosome lengths were displayed in Mbp (megabase pairs) on the scale at top.

In *B. rapa*, 20.34% (60 genes) of the mapped RLKs were found to be organised in 13 gene clusters, which was higher than 10 clusters grouped by 33 genes (13.98%) in the *B. juncea* A genome (Table 3 and Table S1). Furthermore, the cluster distribution of RLKs and RLPs differed between *B. juncea* and *B. rapa* (Figure 7 and Figure 8; Table 3, Tables S1 and S2). For instance, the RLP clusters in the *B. juncea* A genome were evenly located on chromosomes A03, A07 and A08, while they were equally distributed on A05 and A06 of *B. rapa*.

The identification of orthologous genes was performed between *B. juncea* and its diploid progenitors by comparing the protein sequences in each RLK and RLP family, respectively. In *B. juncea*, a total of 383 RLKs (83.62%) and 89 RLPs (41.98%) were considered as orthologous genes, of which 194 RLKs and 24 RLPs were mapped in the A genome, and the remaining genes were distributed in the B genome (Table 4 and Table 6). The orthology analysis also indicated that there were 214 RLKs (72.54%) and 27 RLPs (41.54%) conserved in *B. rapa* and 227 RLKs and 72 RLPs maintained in *B. nigra*, with 71.61% and 40.91%, respectively (Table 3, Table 4, Table 5 and Table 6). In these *Brassica* species, the proportion of conserved RLK genes was higher than RLP genes.

Genomic distribution of orthologous genes indicated that the proportion of conserved RLKs in each sub-genome of *B. juncea* (82.20% and 85.14%) was higher than *B. rapa* (72.54%) and *B. nigra* (71.61%), respectively (Table 3, Table 4, Table 5 and Table 6). Similarly, the percentage of conserved RLPs was also higher in the B genome of *B. juncea* (55.56%) than its progenitor *B. nigra* (40.91%), while obviously lower in the A genome of *B. juncea* than *B. rapa*, with 25.26% and 41.54%, respectively (Table 3, Table 4, Table 5 and Table 6).

In *B. juncea*, 86 RLKs and 38 RLPs were found to be lost in the A genome, and 90 RLKs and 104 RLPs were absent in the B genome, compared to its diploid progenitors (Table 6). Additionally, some RLKs and RLPs were only observed in *B. juncea*, including 42 RLKs and 71 RLPs in the A genome and 33 RLKs and 52 RLPs in the B genome (Table 6).

The synteny relationship of RLK and RLP genes in the A genome was assessed between *B. juncea* and its diploid progenitor *B. rapa* on the basis of an orthologous analysis and their physical positions. This analysis, which was separately performed in the RLK and RLP families, indicated that the distribution of RLKs and RLPs displayed obvious synteny and a collinear relationship between the A genomes of *B. juncea* and *B. rapa* (Figure 7 and Figure 8). In the *B. juncea* A genome, a total of 192 RLKs (81.36%) were found to be in synteny and collinear with *B. rapa* (Table 4). However, only 28.42% (27 genes) of the total RLP genes in the *B. juncea* A genome were observed to be in synteny and collinear with *B. rapa* (Table 4).

## 4. Discussion

### 4.1. Genome Wide Identification of RLK and RLP Genes in B. juncea

The number of LRR-RLKs in *B. juncea* is notably higher than the number in *B. rapa* with 303 genes [52], *A. thaliana* containing 213 genes [54], *O. sativa* having 309 [55] and *P. trichocarpa* having 379 [56]. This is probably due to the large allotetraploid genome of *B. juncea* (AABB) [33]. Similarly, a higher number of LRR-RLKs has also been identified in polyploid *T. aestivum* and *G. max*, with 531 and 467 genes, respectively [48,57,58,59], and allotetraploid *B. napus* cv. Darmor V4 (596 genes) [60]. Accordingly, the LRR-RLP number in *B. juncea* is also higher than in the diploids *B. rapa* and *B. nigra* analysed in this study and the plants previously reported, including *A. thaliana* (57) [12], *O. sativa* (90) [21] and *P. trichocarpa* (82) [20].

Here, the content of LysM of RLKs and RLPs is extremely low in the studied species. Similarly, the number of LysM-RLKs and LysM-RLPs is also very low in previous reports across species from the Brassicaceae family [60]. To date, the highest number (17) of LysM-RLKs have been separately observed in *L. japonicas* and *M. truncatula* [61,62], followed by *G. max* and *P. trichocarpa* with 12 and 11 genes, respectively [63], while less than 10 genes have been observed in *A. thaliana* (5 genes) and *O. sativa* (6 genes) [62,63]. For LysM-RLPs, there are three genes found in *A. thaliana*, [64,65], four in *G. max*, two in *M. truncatula*, seven in *P. trichocarpa* and six in *O. sativa* [65].

The distribution of RLKs and RLPs on the majority of chromosomes was uneven. The distribution pattern of LRR-RLK in *B. rapa*, detected in this study, is consistent with the previous report [52]. The uneven distribution of LRR-RLKs is not novel in plants’ genomes [18], as has been demonstrated in other plant species recently, including *S. lycopersicum* [46], *T. aestivum* [48], two Glycine species (*G. max* and *G. latifolia*) [57,66] and two Citrus species (*C. clementina* and *C. sinensis*) [67]. While the genome-wide study of LRR-RLPs is limited in several plant species, only *O. sativa* and *Arabidopsis* had their distribution analysed, where they exhibit a similar pattern [21] compared with our study. Additionally, the unequal distribution of genes across sub-genomes was also observed in other *Brassica* gene families, such as the NLR family in *B. juncea* [41], *B. napus* [45] and *B. rapa* [68] and the chitinase family in *B. rapa* [69]. Moreover, in this study, many RLKs and RLPs were grouped within the gene clusters. The formation of resistance-gene clusters is thought to facilitate the evolution of resistance genes through sequence exchanges via unequal crossing over, recombinatorial mispairing, or generating high haplotypic diversity [70,71].

### 4.2. RLK and RLP Gene Duplication in B. juncea

Gene duplication, as a result of whole-genome duplication, segmental duplication, tandem duplication and transposition events, is believed to contribute significantly to evolutionary innovation and gene family expansion, which allow the plants to be “buffered” against selective pressure in natural environments [72,73].

In this study, approximately 90% of the assigned RLKs have been found to be duplicated, suggesting that gene duplications contribute significantly to enlarge the RLK family in *B. juncea*. Although the duplicated proportion of RLPs is lower than RLKs, the 53% duplication rate indicates that gene duplication also plays major roles in the expansion of *B, juncea* RLPs. Only around 4% of duplications in both the RLK and RLP families is considered as segmental duplications, suggesting that segmental duplications contribute minimally to the expansion of RLKs and RLPs. However, tandem duplications are likely to be mainly responsible for RLP duplications, due to around 50% duplications being defined as tandem duplications, yet they play minor roles in RLK duplications. Tandem duplication has been reported to be mainly responsible for the expansion of the LRR-RLK subfamily in *O. sativa* [55,74] and five Rosaceae species [75], as well as two additional *O. sativa* gene families, RLP and NLR [21,76]. However, this kind of duplication plays a minor role to enlarge the LRR-RLK sub-family in *A. thaliana*, *G. max* and *S. lycopersicum*, with around 15%, 20.3% and 12% of LRR-RLKs being tandemly duplicated, respectively [46,57,74]. In *P. trichocarpa* and *G. max*, the explosion of LRR-RLKs is significantly caused by segmental duplications, with approximately 82% and 73.3%, respectively [56,57], compared with about 11% in *O. sativa* and around 26% in *A. thaliana* [74]. Additionally, these two major mechanisms proved to play primary roles in the expansion of LRR-RLK subfamily in *B. rapa* and RLK/Pelle family in *A. thaliana* [22,52].

In the *Brassica* species, all of the genomes have undergone a lineage-specific whole-genome triplication. The number of genes increased after many rounds of duplication [77,78,79,80]. Thus, we speculate that the expansion of RLK and RLP genes is probably mainly caused by whole-genome duplication. After speciation, *B. juncea* underwent substantial genome reshuffling and chromosomal rearrangements [33,81,82,83], which probably resulted in the random distribution of duplicated RLKs and RLPs across the *B. juncea* genome.

Here, 37.50% RLK clusters and 85.71% RLP clusters in *B. juncea* were generated by tandemly duplicated genes, indicating that tandem duplication events should be responsible for the origin of the genes in each cluster, which also have been proved in some species, including monocots *A. trichopoda* and *O. sativa* [55,84], eudicots *P. trichocarpa* and *G. max* [56,57]. Some inter-genomic duplications are likely to be from homoeologous regions and the duplication is due to polyploidization.

### 4.3. Phylogenetic Analysis

In this study, the phylogenetic trees were generated using full-length protein sequences of RLKs and RLPs. Full protein sequences of LRR-RLKs, which can provide excellent evolutionary inference, have been used for phylogenetic analysis with the neighbour-joining (NJ) method in many plants [48,52]. In both the RLK and RLP families, the predicted paralogous sequences, which are derived by gene duplications, are closely grouped together on the same or a close clade. Similarly, this has been observed in *A. thaliana*, *B. rapa*, *O. sativa*, *P. trichocarpa* and *T. aestivum*, due to paralogous sequences generated by duplication events and having similar domain architecture [21,48,52]. This number of paralogous sequences indicates that gene duplication plays an important role in the expansion of this family. The divergence of the sequences may be consistent with divergence in function of the genes in these families, as they are known to play roles in a large number of processes and not just disease resistance. Linking the genes involved in disease to specific clades may aid in the identification of candidate genes.

In phylogenetic analysis, protein sequences of the RLP family have formed many distinct clades constructed by branches with a single gene, compared to RLKs. This indicates that RLPs have undergone faster evolution and maintained relatively more diversity in sequence than RLKs, due to greater selective pressure on the RLPs. This phenomenon is also shown in the NLR family, of which the CNL family is more highly diverged than the TNL family in the Cucurbitaceae, Fabaceae, Brassicaceae, Poaceae and Solanaceae species [45,76,85,86].

### 4.4. Comparative Analysis of RLK and RLP Genes in B. juncea to Its Related Diploid Progenitor Species B. rapa and B. nigra

The comparison between *B. juncea* and its progenitors (*B. rapa* and *B. nigra*) reveals RLKs and RLPs are reduced in *B. juncea*, probably due to gene loss during the polyploidization events. This was also observed in the *B. juncea* NLR family [41]. The gene reduction of NLR family has also been reported in *B. napus*, compared with its progenitors *B. rapa* and *B. oleracea* [45]. Additionally, LRR-*RLK* gene reduction has been observed in other species. For example, *Triticum aestivum* (wheat), an allohexaploid species with AABBDD genome, was found to contain 531 LRR-RLKs, distributed in the A, B and D sub-genomes, with 166, 195 and 170 genes, respectively, while the gene number of A and D sub-genomes is lower than its progenitors *Triticum urartu* (AA, 217 genes) and *Aegilops tauschii* (DD, 248 genes) [48,58,87,88,89].

The proportions of LRR-RLK genes in *B. juncea*, *B. rapa* and *B. nigra* are consistent with estimates of LRR-RLK for other angiosperm species, ranging from 0.67% to 1.39% [90]. Therefore, the gene number of LRR-RLKs seems not to be correlated with genome size, which can also be observed in LysM-RLKs and RLPs in this study. Similarly, the gene number of NLRs seems to be nonlinearly related with the total predicted gene number, and is unproportional to genome size in *B. napus*, *B. rapa* and *B. oleracea* [45,91]. It has been reported that a total of 303 LRR-RLK genes were identified in *B. rapa* [52], 10 genes more than that found in the present study. Thus, the difference of gene number is likely caused by using different detection criteria and different reference versions of *B. rapa* genome, here we used *B. rapa* v2.5 updated by Cai, Wang, Liu, Wu, Liang, Cui, Cheng and Wang [42].

The RLPs and RLKs in the A genome of *B. juncea* are consistent with *B. rapa* in their distribution and synteny. In particular, the gene families are highly conserved in size and orientation, similar to the *B. napus* NLR family [45]. The gene conservation and syntenic relationship has been found previously in many plants, such as *G. max*, *A. thaliana* and *B. nigra*, Solanaceae species, *T. urartu* and *T. aestivum* [92,93,94,95,96,97].

Furthermore, significant genomic co-linearity has been reported between *B. napus* and its diploid progenitors *B. rapa* and *B. oleracea* [98,99]. Therefore, we speculate that RLKs and RLPs in the *B. juncea* B genome are also syntenic to *B. nigra*, although a high quality genome assembly of *B. nigra* is not yet available for comparison. However, the absence of synteny in some gene clusters and the presence of non-orthologous genes, which have been observed in the A genome of *B. juncea* in contrast with *B. rapa*, suggest occurrences of gene deletion, translocation and divergence in the *B. juncea* A genome after speciation. This was also reported in the NLR gene family between *B. napus* and its progenitors [45].

Orthology analysis indicates that over 82% RLKs are found to be conserved and maintained in *B. juncea*, compared to *B. rapa* and *B. nigra*. For RLP genes, the conserved proportion is consistent between the B genome of *B. juncea* and *B. nigra*, whereas the RLP conserved proportion is much lower in the *B. juncea* A genome than its progenitor *B. rapa*. This indicates that RLPs in the A genome are more diverse than the in the B genome. The presence of orthologous genes suggests these genes have been retained after genome polyploidization. In plants, disease resistance genes, such as NLR, RLK and RLP families are highly duplicated and have undergone diversifying selection compared to other developmental genes [21,100,101]. Thus, these orthologous copies, which are found to be located in syntenic region between *B. juncea* and its progenitors, probably maintain their original function in the growth and development of *B. juncea*. Non-orthologous genes in *B. juncea* are likely to be specific or gained novel disease resistance.

The gene number of RLKs and RLPs varies within species due to gene losses and/or expansion through duplication and/or divergence [48,67,75,84,90,101]. Moreover, gene loss and gain was reported in *B. juncea* previously, compared to its related diploids [33]. In this study, the differences in gene number of conserved RLKs and RLPs between *B. juncea* and its diploid progenitors indicates the gene loss and gain in *B. juncea*. Reduction in RLKs and RLPs, which is primarily caused by gene loss during polyploidizations, was observed in *B. juncea*, compared to *B. rapa* and *B. nigra*. Simultaneously, gene duplication and recombination of RLKs and RLPs does not generate novel disease resistance genes specific to *B. juncea*.

In polyploids, the duplicated disease resistance genes were found to be preferentially lost after genome duplication, and most of the gene losses were probably driven by a deletion mechanism [102]. In *B. juncea*, the gene loss of RLKs and RLPs are likely caused by species-specific gene deletion after natural hybridisation of *B. juncea* from *B. rapa* and *B. nigra*. A similar kind of deletion was also reported in *T. aestivum* gene families: LRR-RLK, lectin receptor kinases and Glyceraldehyde-3-phosphate dehydrogenase [48,49,103], and the NLR family in *B. napus* [45].

In summary, a notable deletion of RLKs and RLPs was not observed in *B. juncea*, compared to its diploid progenitors, *B. rapa* and *B. nigra*. Evolutionary selection may play a minor role in maintaining the properties of *B. juncea* RLKs, compared with *B. rapa* and *B. nigra*, while being responsible for the diversity of *B. juncea* RLP genes.

## 5. Conclusions

In this study, the sub-families of *B. juncea* LRR-RLK and LRR-RLP are found to be larger than those in most reported plants. Furthermore, most of the RLK and RLP genes are not grouped within the clusters (singletons). Moreover, the occurrence of numerous paralogous genes of RLKs and RLPs indicates that gene duplications significantly contribute to the expansion of RLK and RLP families during the evolution process. Additionally, tandem duplications play major roles in RLP duplications, while segmental duplications make limited contributions to RLK and RLP duplications.

The comparative genome analysis indicates that gene loss and gain of RLKs and RLPs are observed in *B. juncea*, compared to its diploid progenitors, *B. rapa* and *B. nigra*. Furthermore, RLK and RLP genes show syntenic relationships with *B. rapa* in the A genome. The presence of orthologous genes suggests the remaining of conserved RLKs and RLPs after *B. juncea* speciation. However, RLPs are likely to have a faster evolution than RLKs.

The identification and characterisation of these genes will provide a useful resource for *Brassica* researchers undertaking the identification of resistance genes for *Brassica* crop improvement or for evolutionary studies.

## Figures and Tables

**Table 1:** The total number of predicted receptor-like kinase (RLK) and receptor-like protein (RLP) genes identified in the *Brassica* genomes.

Species	*B. juncea*	*B. rapa*	*B. nigra*
Gene content	80,430	46,098	49,826
RLKs	493 (0.613%)	300 (0.651%)	317 (0.636%)
LRR-RLKs	484 (0.602%)	297 (0.644%)	312 (0.626%)
LysM-RLKs	9 (0.011%)	3 (0.007%)	5 (0.010%)
RLPs	228 (0.283%)	65 (0.141%)	176 (0.353%)
LRR-RLPs	226 (0.281%)	63 (0.137%)	175 (0.351%)
LysM-RLPs	2 (0.002%)	2 (0.004%)	1 (0.002%)

**Table 2:** The duplication events defined in the *B. juncea* genome.

Duplication Type	RLK				RLP			
	Total	Intra Sub-Family		Inter Sub-Family	Total	Intra Sub-Family		Inter Sub-Family
		LysM-	LRR-			LysM-	LRR-	
Intra A genomic	83	1	82	0	20	0	20	0
Intra B genomic	80	0	80	0	32	0	32	0
Total of intra genome	163	1	162	0	52	0	52	0
Inter genome	308	4	304	0	58	1	57	0
Total	471	5	466	0	110	1	109	0

**Table 3:** The total number of candidate *RLK* and *RLP* genes identified in *B. rapa*. The total number of genes in each chromosome, as well as the conserved genes obtained from the comparison with *B. juncea*.

Chromosome		RLK								RLP							
		Total	LysM-RLK	LRR-RLK	Gene Cluster		Conserved			Total	LysM-RLP	LRR-RLP	Gene Cluster		Conserved		
					No. of Cluster	No. of Gene in Cluster	Total	LysM-RLK	LRR-RLK				No. of Cluster	No. of Gene in Cluster	Total	LysM-RLP	LRR-RLP
A genome *B. rapa*	A01	36	0	36	1	5	29	0	29	10	0	10	0	0	5	0	5
	A02	28	0	28	2	9	17	0	17	7	0	7	0	0	2	0	2
	A03	38	0	38	0	0	30	0	30	9	0	9	0	0	2	0	2
	A04	16	1	15	1	5	14	1	13	1	0	1	0	0	1	0	1
	A05	25	0	25	1	7	19	0	19	6	0	6	1	3	4	0	4
	A06	49	1	48	3	15	36	1	35	10	1	9	1	3	2	1	1
	A07	37	0	37	3	12	21	0	21	4	0	4	0	0	2	0	2
	A08	19	1	18	0	0	15	1	14	7	1	6	0	0	4	1	3
	A09	26	0	26	0	0	16	0	16	9	0	9	0	0	4	0	4
	A10	21	0	21	2	7	17	0	17	2	0	2	0	0	1	0	1
Total mapped genes		295	3, 1.02% ^1^	292, 98.98% ^1^	13	60, 20.34% ^1^	214, 72.54% ^1^	3, 100.00% ^2^	211, 72.26% ^3^	65	2, 3.08% ^1^	63, 96.92% ^1^	2	6, 9.23% ^1^	27, 41.54% ^1^	2, 100.00% ^2^	25, 39.68% ^3^
Unassigned		5	0	5	-	-	-	-	-	0	0	0	-	-	-	-	-
Total		300	3, 1.00% ^4^	297, 99.00% ^4^	13	-	-	-	-	65	2, 3.08% ^4^	63, 96.92% ^4^	2	-	-	-	-

^1^ The proportion in all mapped RLKs or RLPs. ^2^ The proportion in mapped LysM-RLKs or LysM-RLPs. ^3^ The proportion in mapped LRR-RLKs or LRR-RLPs. ^4^ The proportion in all candidate RLKs or RLPs.

**Table 4:** The total number of candidate RLK and RLP genes identified in *B. juncea*. The total number of genes in each chromosome, as well as the conserved genes obtained from the comparison with *B. nigra* and *B. rapa*. The total number of syntenic genes in each chromosome of *B. juncea* A genome, in contrast with *B. rapa*.

Chromosome		RLK							RLP						
		Total	LysM-	LRR-	Conserved			Syntenic Genes	Total	LysM	LRR-	Conserved			Syntenic Genes
					Total	LysM-	LRR-					Total	LysM-	LRR-	
A genome *B. juncea*	A01	30	0	30	24	0	24	22	12	0	12	6	0	6	5
	A02	25	0	25	17	0	17	14	5	0	5	2	0	2	2
	A03	29	0	29	26	0	26	28	17	0	17	1	0	1	1
	A04	16	2	14	13	1	12	13	9	0	9	1	0	1	1
	A05	22	1	21	18	0	18	19	6	0	6	1	0	1	3
	A06	31	1	30	27	1	26	32	7	1	6	2	1	1	2
	A07	26	0	26	22	0	22	21	11	0	11	2	0	2	2
	A08	12	1	11	12	1	11	11	8	0	8	3	0	3	5
	A09	23	0	23	17	0	17	15	17	0	17	5	0	5	5
	A10	22	0	22	18	0	18	17	3	0	3	1	0	1	1
Total in A genome		236, 51.53% ^1^	5, 2.12% ^2^	231, 97.88% ^2^	194, 82.20% ^2^	3, 60.00% ^3^	191, 82.68% ^4^	192, 81.36% ^2^	95, 44.81% ^1^	1, 1.05% ^2^	94, 98.95% ^2^	24, 25.26% ^2^	1, 100.00% ^3^	23, 24.47% ^4^	27, 28.42% ^2^
B genome *B. juncea*	B01	27	2	25	24	2	22	-	6	0	6	6	0	6	-
	B02	36	0	36	30	0	30	-	10	0	10	3	0	3	-
	B03	30	0	30	27	0	27	-	19	1	18	13	1	12	-
	B04	30	1	29	23	1	22	-	23	0	23	11	0	11	-
	B05	33	0	33	28	0	28	-	18	0	18	11	0	11	-
	B06	20	0	20	17	0	17	-	11	0	11	6	0	6	-
	B07	16	0	16	16	0	16	-	16	0	16	9	0	9	-
	B08	30	0	30	24	0	24	-	14	0	14	6	0	6	-
Total in B genome		222, 48.47% ^1^	3, 1.35% ^2^	219, 98.65% ^2^	189, 85.14% ^2^	3, 100.00% ^3^	186, 84.93% ^4^	-	117, 55.19% ^1^	1, 0.85% ^2^	116, 99.15% ^2^	65, 55.56% ^2^	1, 100.00% ^3^	64, 55.17% ^4^	-
Total mapped genes		458	8, 1.75% ^1^	450, 98.25% ^1^	383, 83.62% ^1^	6, 75.00% ^3^	377, 83.78% ^4^	-	212	2, 0.94% ^1^	210, 99.06% ^1^	89, 41.98% ^1^	2, 100.00% ^3^	87, 41.43% ^4^	-

^1^ The proportion in all mapped RLKs or RLPs. ^2^ The proportion in all mapped RLKs or RLPs in each sub-genome of *B. juncea*. ^3^ The proportion in mapped LysM-RLKs or LysM-RLPs across the entire genome and each sub-genome of *B. juncea*. ^4^ The proportion in mapped LRR-RLKs or LRR-RLPs across the entire genome and each sub-genome of *B. juncea*.

**Table 5:** The total number of candidate RLK and RLP genes identified in *B. nigra* scaffolds. The conserved genes obtained from the comparison with *B. juncea* and *B. nigra*.

**RLK**	Total		317
	LysM-RLK		5 (1.58% ^1^)
	LRR-RLK		312 (98.42% ^1^)
	Conserved	Total	227 (71.61% ^1^)
		LysM-RLK	4
		LRR-RLK	223
**RLP**	Total		176
	LysM-RLP		1 (0.57% ^2^)
	LRR-RLP		175 (99.43% ^2^)
	Conserved	Total	72 (40.91% ^2^)
		LysM-RLP	1
		LRR-RLP	71

^1^ The proportion in all RLKs. ^2^ The proportion in all RLPs.

**Table 6:** Comparative analysis of the number of RLK and RLP genes in *B. juncea*, *B. nigra* and *B. rapa*.

Gene Family	Genome	*B. juncea* A Genome	*B. rapa* A Genome	*B. juncea* B Genome	*B. nigra* B Genome
RLK family	Total (LysM, LRR)	236 (5, 231)	300 (3, 297)	222 (3, 219)	317 (5, 315)
	Total conserved	194 (82.20%)	214 (71.33%)	189 (85.14%)	227 (71.61%)
	Conserved LysM-RLK	3 (60.00%)	3 (100.00%)	3 (100.00%)	4 (80.00%)
	Conserved LRR-RLK	191 (82.68%)	211 (71.04%)	186 (84.93%)	223 (70.79%)
	Not conserved	42 (17.80%)	86 (28.67%)	33 (14.86%)	90 (28.39%)
	Lost	0	86	0	90
	Gained	42	0	33	0
RLP family	Total (LysM, LRR)	95 (1, 94)	65 (2, 63)	117 (1, 116)	176 (1, 175)
	Total conserved	24 (25.26%)	27 (41.54%)	65 (55.56%)	72 (40.91%)
	Conserved LysM-RLP	1 (100.00%)	2 (100.00%)	1 (100.00%)	1 (100.00%)
	Conserved LRR-RLP	23 (24.47%)	25 (39.68%)	64 (55.17%)	71 (40.57%)
	Not conserved	71 (74.74%)	38 (58.46%)	52 (44.44%)	104 (59.09%)
	Lost	0	38	0	104
	Gained	71	0	52	0

## Data Availability

The data presented in this study are available in supplementary Table S1.

## Supplementary Materials

The following are available online at https://www.mdpi.com/2079-7737/10/1/17/s1, Figure S1: Phylogenetic analysis of RLK proteins of *B. juncea*, Figure S2: Phylogenetic analysis of RLP proteins of *B. juncea*, Table S1: The distribution and organisation of candidate RLK genes in *B. juncea*, Table S2: The distribution and organisation of candidate RLP genes in *B. juncea*.
