# Supplementary material for: Genome-Wide Identification and Evolution of Receptor-Like Kinases (RLKs) and Receptor like Proteins (RLPs) in Brassica juncea
Source: Biology (Basel). 2020 Dec 30;10(1):17. doi: 10.3390/biology10010017 (PMC7823396; doi:10.3390/biology10010017)
Supplement: Supplementary file 1 [file biology-10-00017-s001.pdf]

biology

MDPI

Supplementary Materials

Genome-Wide Identification and Evolution of Receptor-Like Kinases (RLKs) and Receptor like Proteins (RLPs) in *Brassica juncea*

Hua Yang, Philipp E. Bayer, Soodeh Tirnaz, David Edwards and Jacqueline Batley

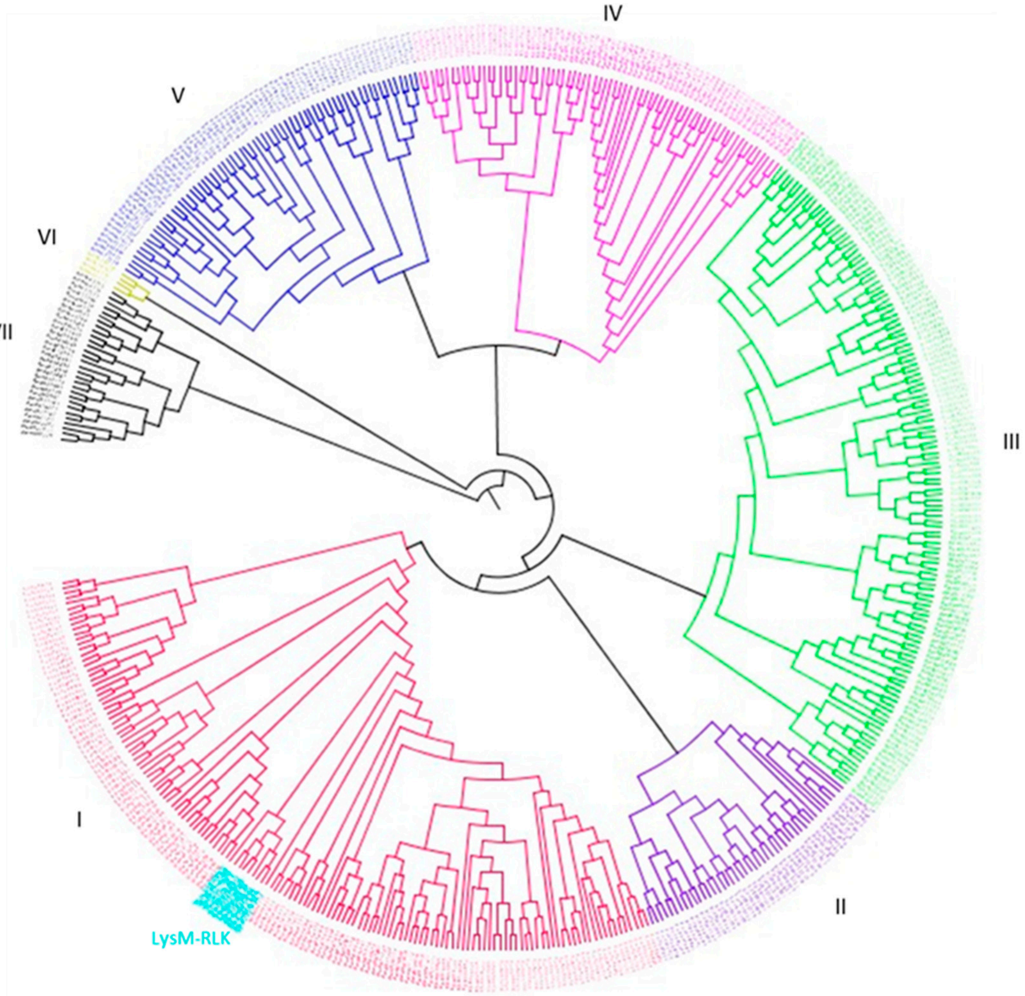

Figure S1. Phylogenetic analysis of RLK proteins of *B. juncea*. The circular tree was generated based on the neighbour-joining method using Geneious v7.1.9 software. These genes were divided into 7 main groups by different colours. In Group I, labels highlighted with light-blue were LysM-RLK genes.

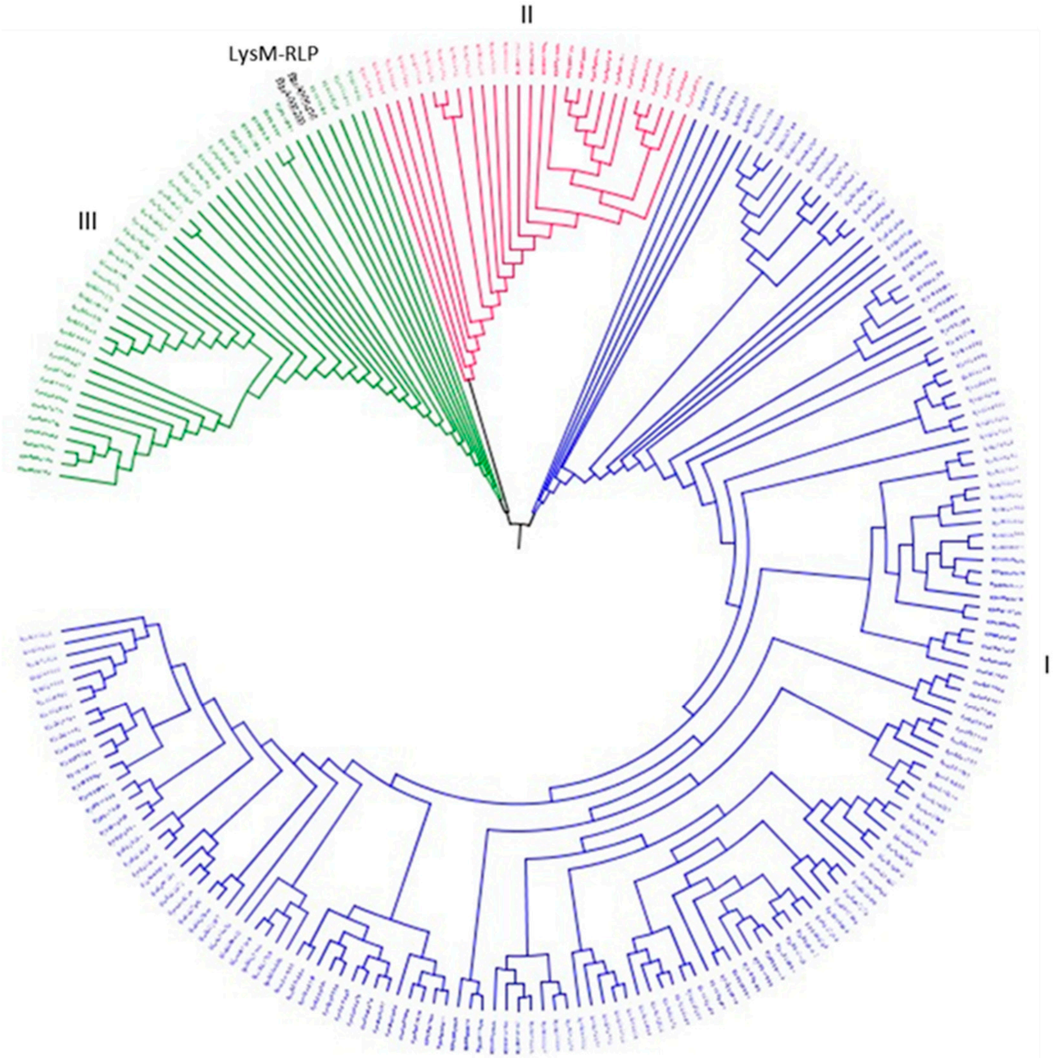

Figure S2. Phylogenetic analysis of RLP proteins of *B. juncea*. The circular tree was generated based on the neighbour-joining method using Geneious v7.1.9 software. These genes were divided into 3 main groups by different colours. In Group III, labels highlighted with black were LysM-RLP genes.

Table S1. The distribution and organisation of candidate RLK genes in *B. juncea.*

Genome	Chromosome	Total	LysM-RLK	LRR-RLK	No. of gene cluster (tandem cluster)	No. of gene in clusters			No. of duplicated genes			No. of duplicated genes in clusters			Tandem duplication genes	Segmental duplication genes	Duplication type	
						Total (in each cluster)	LysM-RLK	LRR-RLK	Total	LysM-RLK	LRR-RLK	Total (tandem duplications)	LysM-RLK	LRR-RLK			Intra-genomic	Inter-genomic
A genome *B. juncea*	A01	30	0	30	1	4 (4)	0	4	25	0	25	3	0	3	0	0	√	√
	A02	25	0	25	2 (1)	6 (3+3)	0	6	19	0	19	3 (2)	0	3	4	0	√	√
	A03	29	0	29	1 (1)	3 (3)	0	3	25	0	25	2 (2)	0	2	2	0	√	√
	A04	16	2	14	0	0	0	0	15	2	13	0	0	0	2	0	√	√
	A05	22	1	21	1	4 (4)	0	4	21	0	21	4	0	4	0	2	√	√
	A06	31	1	30	2 (1)	7 (4+3)	1	6	29	1	28	7 (2)	1	6	12	4	√	√
	A07	26	0	26	0	0	0	0	22	0	22	0	0	0	0	2	√	√
	A08	12	1	11	0	0	0	0	11	1	10	0	0	0	0	0	√	√
	A09	23	0	23	1	3 (3)	0	3	22	0	22	3	0	3	0	0	√	√
	A10	22	0	22	2	6 (3+3)	0	6	20	0	20	5	0	5	2	0	√	√
	Total	236, 51.53% [1]	5	231	10 (3)	33	1	32	209	4	205	27 (6)	1	26	22	8	-	-
B genome *B. juncea*	B01	27	2	25	0	0	0	0	26	2	24	0	0	0	2	0	√	√
	B02	36	0	36	1	5 (5)	0	5	34	0	34	5	0	5	0	2	√	√
	B03	30	0	30	1	4 (4)	0	4	26	0	26	3	0	3	6	2	√	√
	B04	30	1	29	1	6 (6)	0	6	25	1	24	3	0	3	10	0	√	√
	B05	33	0	33	1 (1)	6 (6)	0	6	32	0	32	5 (4)	0	5	8	0	√	√
	B06	20	0	20	0	0	0	0	17	0	17	0	0	0	0	0	√	√
	B07	16	0	16	0	0	0	0	16	0	16	0	0	0	0	2	√	√
	B08	30	0	30	2 (2)	8 (5+3)	0	8	28	0	28	8 (7)	0	8	10	2	√	√
	Total	222, 48.47% [1]	3	219	6 (3)	29	0	29	204	3	201	24 (11)	0	24	36	8	-	-
Total mapped RLK genes		458, 92.90% [4]	8, 1.75% [1]	450, 98.25% [1]	16 (6)	62, 13.54% [1]	1, 12.50% [2]	61, 13.56% [3]	413, 90.17% [1]	7, 87.50% [2]	406, 90.22% [3]	51 (17)	1	50	58	16	-	-
Unassigned		35, 7.10% [4]	1	34	-	-	-	-	-	-	-	-	-	-	-	-	-	-
Total		493	9, 1.83% [4]	484, 98.17% [4]	-	-	-	-	-	-	-	-	-	-	-	-	-	-

[1] The proportion in all mapped RLK genes. [2] The proportion in all mapped LysM-RLK genes. [3] The proportion in all mapped LRR-RLK genes. [4] The proportion in all RLK genes.

Table S2. The distribution and organisation of candidate RLP genes in *B. juncea.*

Genome	Chromosome	Total	LysM-RLP	LRR-RLP	No. of gene cluster (tandem cluster)	No. of gene in clusters			No. of duplicated genes			No. of duplicated genes in clusters			Tandem duplication genes	Segmental duplication genes	Duplication type	
						Total	LysM-RLP	LRR-RLP	Total	LysM-RLP	LRR-RLP	Total	LysM-RLP	LRR-RLP			Intra-genomic	Inter-genomic
A genome *B. juncea*	A01	12	0	12	0	0	0	0	7	0	7	0	0	0	0	0	√	√
	A02	5	0	5	0	0	0	0	1	0	1	0	0	0	0	0	×	√
	A03	17	0	17	1 (1)	3	0	3	10	0	10	3 (3)	0	3	10	0	√	√
	A04	9	0	9	0	0	0	0	5	0	5	0	0	0	2	0	√	√
	A05	6	0	6	0	0	0	0	1	0	1	0	0	0	0	0	√	×
	A06	7	1	6	0	0	0	0	4	1	3	0	0	0	0	0	×	√
	A07	11	0	11	1 (1)	3	0	3	7	0	7	2 (2)	0	2	2	0	√	√
	A08	8	0	8	1 (1)	4	0	4	7	0	7	4 (3)	0	4	12	0	√	√
	A09	17	0	17	0	0	0	0	9	0	9	0	0	0	2	0	√	√
	A10	3	0	3	0	0	0	0	1	0	1	0	0	0	0	0	√	×
	Total	95, 44.81% [1]	1	94	3 (3)	10	0	10	52	1	51	9 (8)	0	9	28	0	-	-
B genome *B. juncea*	B01	6	0	6	0	0	0	0	4	0	4	0	0	0	0	0	√	√
	B02	10	0	10	0	0	0	0	4	0	4	0	0	0	0	0	√	√
	B03	19	1	18	1 (1)	3	0	3	11	1	10	3 (2)	0	3	6	4	√	√
	B04	23	0	23	1 (1)	5	0	5	11	0	11	5 (4)	0	5	12	0	√	√
	B05	18	0	18	1 (1)	4	0	4	9	0	9	3 (2)	0	3	2	0	√	√
	B06	11	0	11	1	3	0	3	8	0	8	2	0	2	2	0	√	√
	B07	16	0	16	0	0	0	0	10	0	10	0	0	0	2	0	√	√
	B08	14	0	14	0	0	0	0	3	0	3	0	0	0	0	0	√	√
	Total	117, 55.19% [1]	1	116	4 (3)	15	0	15	60	1	59	13 (8)	0	13	24	4	-	-
Total mapped RLP genes		212, 92.98% [4]	2, 0.94% [1]	210, 99.06% [1]	7 (6)	25, 11.79% [1]	0	25, 11.90% [3]	112, 52.83% [1]	2, 100.00% [2]	110, 52.38% [3]	22 (16)	0	22	52	4	-	-
Unassigned		16, 7.02% [4]	0	16	-	-	-	-	-	-	-	-	-	-	-	-	-	-
Total		228	2, 0.88% [4]	226, 99.12% [4]	-	-	-	-	-	-	-	-	-	-	-	-	-	-

[1] The proportion in all mapped RLP genes. [2] The proportion in all mapped LysM-RLP genes. [3] The proportion in all mapped LRR-RLP genes. [4] The proportion in all RLP genes.
